# Radiomics analysis of patellofemoral joint improves knee replacement risk prediction: Data from the Multicenter Osteoarthritis Study (MOST)

**DOI:** 10.1016/j.ocarto.2024.100448

**Published:** 2024-02-24

**Authors:** Jiang Zhang, Tianshu Jiang, Lok-Chun Chan, Sing-Hin Lau, Wei Wang, Xinzhi Teng, Ping-Keung Chan, Jing Cai, Chunyi Wen

**Affiliations:** a Department of Health Technology and Informatics, The Hong Kong Polytechnic University, Hong Kong, China; b Department of Biomedical Engineering, The Hong Kong Polytechnic University, Hong Kong, China; c Department of Orthopaedics and Traumatology, The University of Hong Kong, Hong Kong, China; d Research Institute for Smart Ageing, The Hong Kong Polytechnic University, Hong Kong, China

**Keywords:** Patellofemoral joint, Osteoarthritis, Knee replacement, Lateral knee radiograph, Radiomics

## Abstract

**Objective:**

Knee replacement (KR) is the last-resort treatment for knee osteoarthritis. Although radiographic evidence of tibiofemoral joint has been widely adopted for prognostication, patellofemoral joint has gained little attention and may hold additional value for further improvements. We aimed to quantitatively analyse patellofemoral joint through radiomics analysis of lateral view radiographs for improved KR risk prediction.

**Design:**

From the Multicenter Osteoarthritis Study dataset, we retrospectively retrieved the initial-visit lateral left knee radiographs of 2943 patients aged 50 to 79. They were split into training and test cohorts at a 2:1 ratio. A comprehensive set of radiomic features were extracted within the best-performing subregion of patellofemoral joint and combined into a radiomics score (RadScore). A KR risk score, derived from Kellgren-Lawrence grade (KLG) of tibiofemoral joint and RadScore of patellofemoral joint, was developed by multivariate Cox regression and assessed using time-dependent area under receiver operating characteristic curve (AUC).

**Results:**

While patellofemoral osteoarthritis (PFOA) was insignificant during multivariate analysis, RadScore was identified as an independent risk factor (multivariate Cox p-value < 0.001) for KR. The subgroup analysis revealed that RadScore was particularly effective in predicting rapid progressor (KR occurrence before 30 months) among early- (KLG < 2) and mid-stage (KLG ​= ​2) patients. Combining two joints radiographic information, the AUC reached 0.89/0.87 for predicting 60-month KR occurrence.

**Conclusions:**

The RadScore of the patellofemoral joint on lateral radiographs emerges as an independent prognostic factor for improving KR prognosis prediction. The KR risk score could be instrumental in managing progressive knee osteoarthritis interventions.

## 1 Introduction

Knee osteoarthritis (KOA) is a leading cause of chronic pain and disability in older adults [1]. Knee replacement (KR) surgery is often the last resort of treatment for KOA once the joint is rapidly destructed [2,3]. Therefore, early prediction of KOA progression and KR risk is highly desired to enable timely intervention and effective management of disease deterioration. Some studies have predicted KR using demographics and clinical data, highlighting the importance of the Kellgren-Lawrence grading (KLG) in disease prognosis [4,5]. With advancements in medical imaging analysis technology, several studies directly analysed medical images, primarily Magnetic Resonance Imaging (MRI) and X-ray, for KR prediction [[6], [7], [8], [9], [10], [11], [12]]. One study analysed the shape of MRI femur bone from the Osteoarthritis Initiative (OAI) dataset. It proposed a B-score, yielding a modest improvement in KR prediction with an area under receiver operating characteristic curve (AUC) of 0.85 when combined with the KLG compared to 0.83 of KLG alone [6]. Based on the same dataset, a multi-task deep learning-based model has been developed based on posteroanterior (PA) view TF joint radiographs, achieving an AUC of 0.87 for KR prediction [9]. Notably, a recent study comparing MRI and radiographs from both OAI and Multicenter Osteoarthritis Study (MOST) datasets found that, despite the superior soft tissue contrast of MRI, its predictive performance was only marginally better than radiographs (AUC: 0.89 vs. 0.87) [7]. The majority of these studies have exploited the prognostic value of tibiofemoral (TF) joint in KR surgery. However, while advanced algorithms and various imaging modalities have been used for KOA progression and KR risk, performance improvements remain marginal when focusing solely on the TF joint.

In the meantime, a growing body of evidence suggests the prognostic value of the patellofemoral (PF) joint, the other essential compartment of the knee, in KOA progression. PF osteoarthritis (PFOA) is a highly prevalent disease [13,14] that often develops prior to TF osteoarthritis (TFOA) [15] and leads the way in entire knee joint degeneration [16]. Intriguingly, PFOA exhibits a stronger association with KOA symptomatic deterioration than TFOA [17]. Very recently, radiographic PFOA at baseline was associated with a higher likelihood of KR in 10 years [18]. All the evidence suggests that PF joint status may also render high clinical value in early detection and intervention for progressive KOA.

However, technical challenges remain in how to assess PF joint in a reliable and accurate manner for KR risk evaluation. First, there are no standard assessment criteria for PFOA. Most studies directly employed the KLG system, considering both joint space narrowing and the presence of osteophytes on lateral and/or skyline radiographs [14]. Second, a high inter-observer variability on joint spacing narrowing was reported on lateral radiographs due to the interference of overlapping [19]. On the other hand, skyline view radiographs are more effective than lateral X-rays in thoroughly assessing the extent of patellofemoral joint issues. However, they have been less commonly used by surgeons in the past decades, while lateral X-rays are preferred [20]. This preference has probably led to a lack of skyline view radiographs in public datasets such as the Osteoarthritis Initiative (OAI) and the Multicenter Osteoarthritis Study (MOST). MRI provides excellent detail for studying knee degradation, proving valuable in identifying subchondral bone marrow lesions [21] and cartilage defects [22], and thus informing the development of new biomarkers for KOA [23]. However, due to its high cost, limited accessibility, and risk of overdiagnosis, MRI is not deemed suitable for routine clinical diagnosis or as the primary tool for image biomarker development in everyday clinical practice [24,25]. As such, there is a pressing demand to effectively extract radiographic information on PF joint from the widely available lateral knee radiographs and further enhance the KR risk prediction performance.

In this context, radiomics has been identified as a potentially valuable tool in analysing KOA imaging by leveraging high-throughput extraction of quantitative features from medical images [26]. It could capture additional diagnostic or prognostic information that might not be easily perceived through visual inspection, which can serve as a more objective and quantifiable method to forecast disease progression patterns. This methodology has been proven successful in various areas of clinical research, including oncology, neurology, and increasingly in musculoskeletal disorders such as osteoarthritis.

Given the above, the purpose of this study is to characterise the PF joint using radiomics from lateral knee radiographs for KR prognosis and establish a KR risk score by incorporating demographic and two joints’ radiographic information. By leveraging the radiomics approach, we attempted to extract quantitative radiographic information on the PF joint from lateral knee radiographs, which is prognostic and complementary to the existing KLG, thereby enhancing the KR risk prediction performance and improving patient care.

## 2 Method

### 2.1 Data collection

**Fig. 1:**
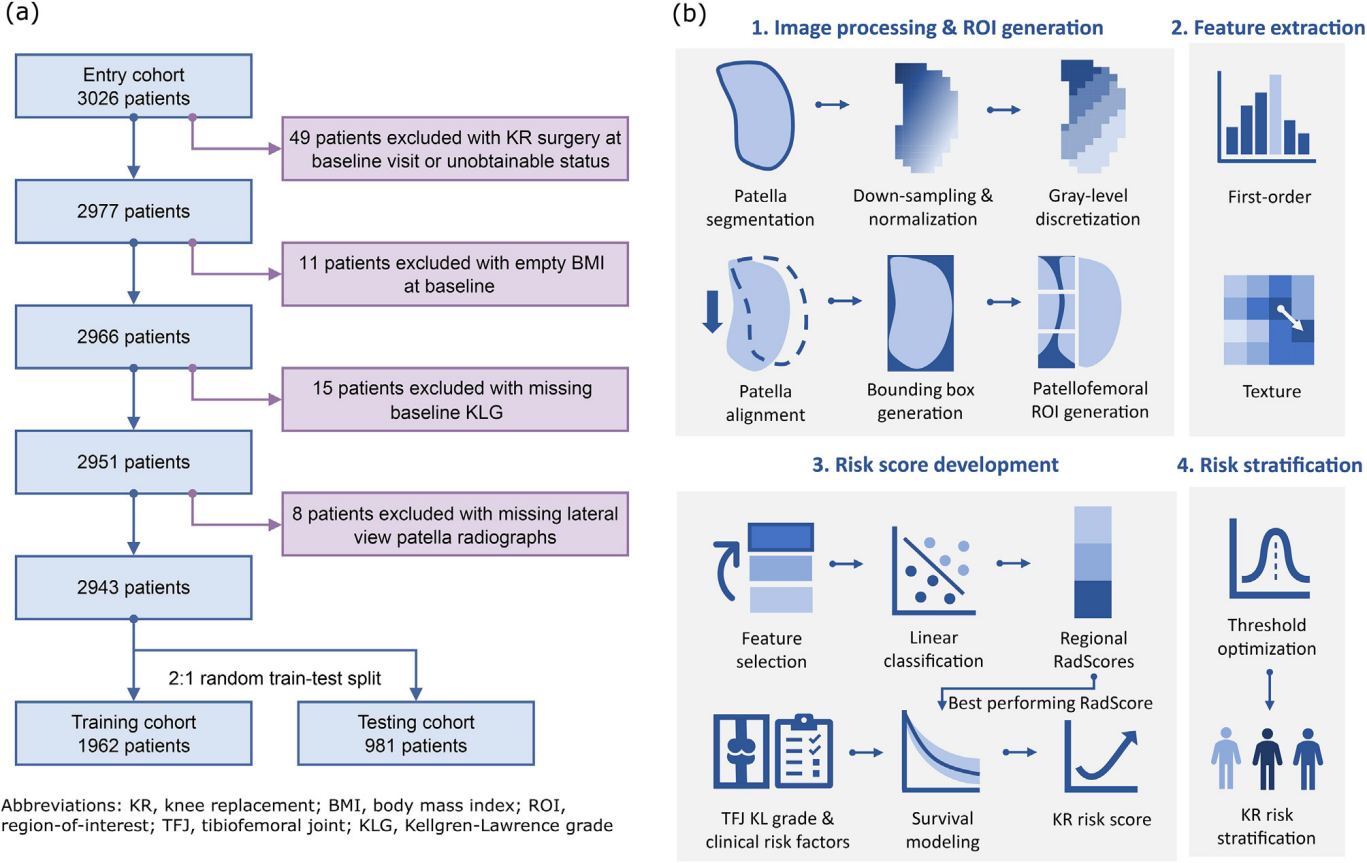
(a) Cohort exclusion criteria and (b) the workflow for knee replacement (KR) risk score development and risk stratification. Tibial-femoral joint Kellgren-Lawrence grade (KLG) was assessed on the anteroposterior view of radiographs. Demographic information includes age, gender, and body mass index (BMI).

A total of 3026 patients aged between 50 and 79 were retrospectively recruited from the MOST dataset [27], with 2943 remaining after applying the exclusion criteria (Fig. 1(a)). Patients were then randomly split (2:1) into one training cohort (n ​= ​1962) and one testing cohort (n ​= ​981). We collected the initial visit left knee weight-bearing lateral radiographs and numerical data, including left knee KLG, KR time and event, and demographic information (age, gender, body mass index (BMI)). Left knee KLG at follow-up visits were also collected. The MOST study was approved by local institutional review boards, and all participants gave written consent at the initial visit. Details about the participants and variable acquisitions can be found in the Supplementary Materials.

### 2.2 Patient and public involvement

It was impossible to involve patients or the public in the design, conduct, reporting, or dissemination plans of our research since it is a secondary analysis of the MOST study.

### 2.3 Study design

Lateral knee radiographs were analysed quantitatively via radiomics, where a large quantity of pre-defined image features was extracted and correlated with KR occurrence at 60-month follow-up. The resulting Radiomics score (RadScore) of PF joint was combined with KLG of TF joint as well as independent prognostic demographic information to generate a comprehensive KR risk score. A three-class risk stratification system was then established based on the KR risk score by optimising two cutoff thresholds targeting different speeds of disease progression. Workflows of KR risk score development and risk stratification are illustrated in Fig. 1(b). The RadScore, KR risk score, and stratification thresholds were solely developed from the training cohort and validated in both the training and testing cohort.

### 2.4 Image processing and region-of-interest (ROI) generation

In order to minimise noise and enhance reproducibility, images were preprocessed by patella alignment, signal enhancement, and noise reduction before feature extraction. First, all patella segmentations were manually drawn by professional radiologists with the Computer Vision Annotation Tool (CVAT). To ensure the vertical alignment of the patella, image orientations were adjusted until the maximum ratio between the vertical and horizontal size of the patella bounding box was reached. Images were first resampled into an isotropic resolution of 0.5 ​mm ​× ​0.5 ​mm to harmonise physical spacing and reduce noise. Contrasts within the patella region were enhanced by Z-score normalisation and thresholding by six standard deviations. The gray levels within the patella bounding box were reduced to 32 bins to further compress the noise.

**Fig. 2:**
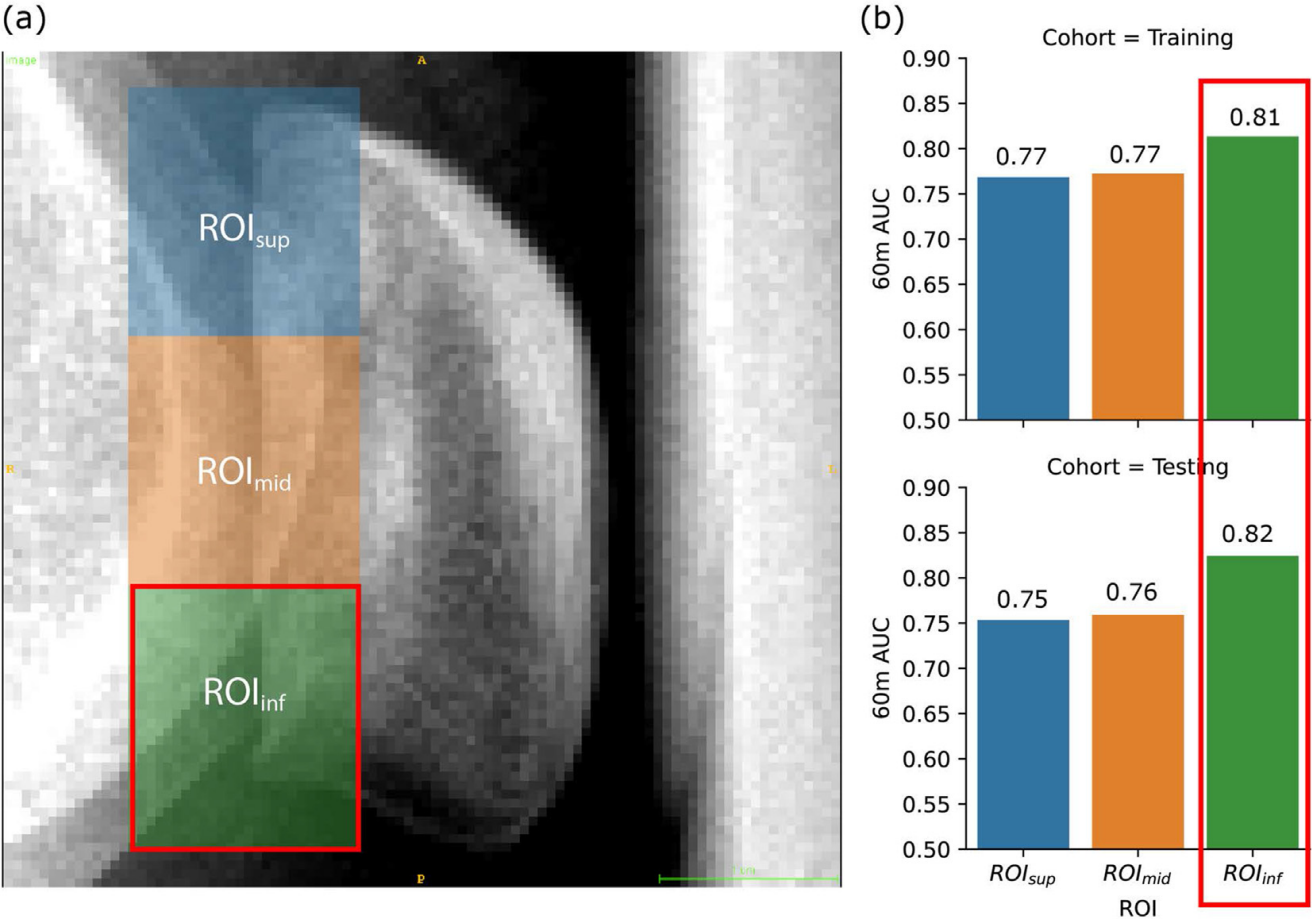
(a) Region-of-interest (ROI) segmentation of one example patient. (b) Bar plot of RadScore AUC of each ROI in prediction 60-month KR (right). ROI_inf_, which is located at the inferior region of the patellofemoral joint and marked by green rectangles, achieved the best performance in both training and testing.

Three equal-sized rectangular regions: ROI_sup_, ROI_mid_, and ROI_inf_ were automatically generated as ROIs to cover three different areas of the PF joint for feature extraction, as demonstrated by an example in Fig. 2(a). They were defined as equal partitions of one-third of the patella bounding box closest to the trochlear area, with a double-size extension towards the posterior direction. This ROI definition was adopted due to its simplicity in clinical application and reduced susceptibility to different observers. All the image processing and ROI generation procedures were performed by the Python package SimpleITK (version 2.2.1) [28].

### 2.5 Feature extraction

A comprehensive set of hand-crafted first-order and texture radiomic features were extracted from each ROI. Specifically, 93 first-order and texture features were calculated following the protocol specified in the Image Biomarker Standardization Initiative (IBSI) [29]. In addition to the original image features, advanced radiomic features were extracted from seven filtered images before gray-level reduction. Both image filtering and radiomic feature extraction were performed by PyRadiomics (version 3.0.0) [30]. In total, 930 radiomics were extracted for each ROI. Detailed settings of the radiomic feature extraction can be found in Supplementary Material Table S1.

### 2.6 RadScore development

Radiomic features were selected separately for each ROI based on inter-feature redundancy and relevancy to 60-month KR to increase the robustness and generalizability of RadScore. They were ranked using the “minimum Redundancy Maximum Relevance” (mRMR) algorithm [31]. The top five features were chosen for RadScore developments.

RadScores were modelled from the selected radiomic features independently for the three ROIs, and the best-performing one was selected as the final RadScore. Binary classification models based on Ridge regression were fitted on 60-month left KR occurrence using the features normalised by Z-score. An easy-ensemble approach was adopted to account for the highly imbalanced distribution of KR, where 500 sub-models were trained under random subsampling and combined to give the final probability prediction. It was performed by the Python package imbalance-learn (version 0.10.1) [32]. The final RadScore was determined as the one with the highest area under the receiver operating characteristic curve (AUC) on 60-month KR occurrence.

### 2.7 KR risk score development and risk stratification

A comprehensive KR risk score was constructed by combining RadScore, KLG, and independently prognostic demographic factors using a multivariate Cox regression model. Patients were stratified into three risk levels based on the KR risk score. The stratification system was designed by optimising two thresholds in classifying.

• Non-progressors: did not receive KR within 84 months (KR- (84 ​m))
• Slow progressors: received KR between 30 and 84 months (KR+ (30 m – 84 ​m))
• Fast progressors: received KR on and before 30 months (KR+ (30 ​m))

The threshold optimisation was performed by maximising Youden's index.

### 2.8 Statistical analyses

In order to evaluate the independent prognostic values of the constructed RadScore, univariate and multivariate Cox regression was used to assess the hazard ratios (HRs) and *p*-values of each KR risk factor. Subgroup analyses were also performed where the time-dependent receiver operating characteristic curves (ROCs) and their AUCs were evaluated within early-stage (KLG < 2), mid-stage (KLG ​= ​2), and late-stage patients (KLG > 2).

The current clinical model that combines the TF and PF joint information was also constructed by joining baseline KLG and PFOA with Cox regression. Performance of the baseline KLG, RadScore, PFOA ​+ ​KLG, and KR risk score were evaluated by concordance index (C-index) and time-dependent ROCs and their AUCs at 30, 60, and 84 months, followed by statistical comparison with the KR risk score. A 95% confidence interval (95CI) was given for each C-index by 1000-iteration bootstrapping. *P*-values for C-index comparisons were calculated by permutation test (one-sided), where the labels were randomly permutated between two groups 1000 times. 95CI estimation and permutation test were performed by Ref. [33] in Python.

The KR risk stratification performance was evaluated by Kaplan-Meier (KM) analysis and further compared with KLG by confusion matrix in classifying the non-, slow, and fast progressors. KM analysis was performed by the Python package lifelines (version 0.27.4) [34].

## 3 Results

### 3.1 Patient characteristics

Distributions of the KR risk factors used in this study and 84-month left KR occurrence were listed in Table 1. Statistically lower baseline BMI (*p*-value ​= ​0.020) was recorded from the training patients compared to testing. The rest of the characteristics, including age, gender, KLG, KR event, and follow-up duration had similar distributions between the two patient cohorts. For patients without KR records in training/testing cohorts, 17/8 patients lost follow-up before 30 months, 42/18 before 60 months, and 79/35 before 84 months.

**Table 1:** Distributions of the included knee replacement risk factors of the training and testing patients.

Parameter	Training	Testing	*p*-value
Patient No.	1962	981	
Age			
Mean	62.42	62.40	0.951
Range	50–79	50–79	
Gender			
Male	1177	587	0.968
Female	785	394	
BMI			
Mean	30.45	30.99	0.020
Range	16.72–57.83	18.50–71.91	
Baseline KLG			
0	882	417	0.251
1	347	165	
2	293	143	
3	294	165	
4	146	91	
Baseline PFOA			
0	1546	755	0.519
1	283	155	
Missing	133	71	
84-month KR			
0	1753	876	1.000
1	209	105	
KR follow-up time			
Medium	84	84	0.536
Range	2–97	3–101	

Note: *p*-values were acquired by Student t-test for continuous variables and nominal variables with > 5 levels, including age, BMI, and KR follow-up time. The rest of the nominal and categorical variables were compared by the Chi-square test.

Abbreviations: KR, knee replacement; BMI, body mass index; KLG, Kellgren and Lawrence grade; PFOA: patellofemoral osteoarthritis.

### 3.2 RadScore composition

Heterogeneous RadScore performances were found among the three ROIs in 60-month KR prediction (Fig. 2(b)). ROI_inf_, covering the inferior PF joint area, reached the highest 60-month KR prediction performance with training and testing AUCs of 0.81 and 0.82, respectively. Therefore, ROI_inf_ was chosen as the ROI for the final RadScore for KR risk score development. Details of the final RadScore compositions can be found in Supplementary Matderial Table S2.

### 3.3 RadScore's independence and predictive value

The PF joint RadScore is an independent risk factor (*p*-value < 0.001) for KR in both univariate and multivariate settings, as reported in Table 2. During the univariate test, all the demographic and radiographic factors were significantly associated with KR in training and testing. However, only KLG and RadScore persisted as independent prognostic factors in both training and testing. Notably, PFOA did not demonstrate independent prognostic value with the presence of RadScore.

**Table 2:** Univariate and multivariate survival analysis results of the final RadScore, baseline KLG, and other knee replacement risk factors in training and testing.

Cohort	Risk factor	Univariate		Multivariate	
		HR (95CI)	*p*-value	HR (95CI)	*p*-value
Training	RadScore	2.49 (2.19–2.83)	**< 0.001**	1.45 (1.21–1.73)	**< 0.001**
	Age	1.04 (1.02–1.05)	**< 0.001**	1.01 (0.99–1.03)	0.270
	Gender	0.74 (0.56–0.99)	**0.046**	0.81 (0.60–1.09)	0.172
	BMI	1.06 (1.04–1.08)	**< 0.001**	0.99 (0.97–1.01)	0.409
	KLG	2.53 (2.25–2.84)	**< 0.001**	1.88 (1.63–2.17)	**< 0.001**
	PFOA	4.72 (3.55–6.26)	**< 0.001**	1.19 (0.85–1.68)	0.304
Testing	RadScore	2.29 (1.91–2.75)	**< 0.001**	1.40(1.09–1.80)	**0.009**
	Age	1.04 (1.01–1.06)	**0.002**	1.00 (0.98–1.03)	0.745
	Gender	0.61 (0.40–0.93)	**0.022**	0.67 (0.43–1.04)	0.073
	BMI	1.05 (1.02–1.07)	**< 0.001**	0.99 (0.96–1.02)	0.682
	KLG	2.39 (2.02–2.82)	**< 0.001**	2.02 (1.65–2.47)	**< 0.001**
	PFOA	4.17 (2.79–6.23)	**< 0.001**	1.12 (0.69–1.83)	0.635

Note: Univariate and multivariate survival analyses were performed by Cox regression. *P*-value less than 0.05 (bolded) was considered significant.

Abbreviations: RadScore, radiomics score; HR, hazard ratio; 95CI, 95% confidence interval; BMI, body mass index; KLG, Kellgren and Lawrence grade; PFOA, patellofemoral osteoarthritis.

**Fig. 3:**
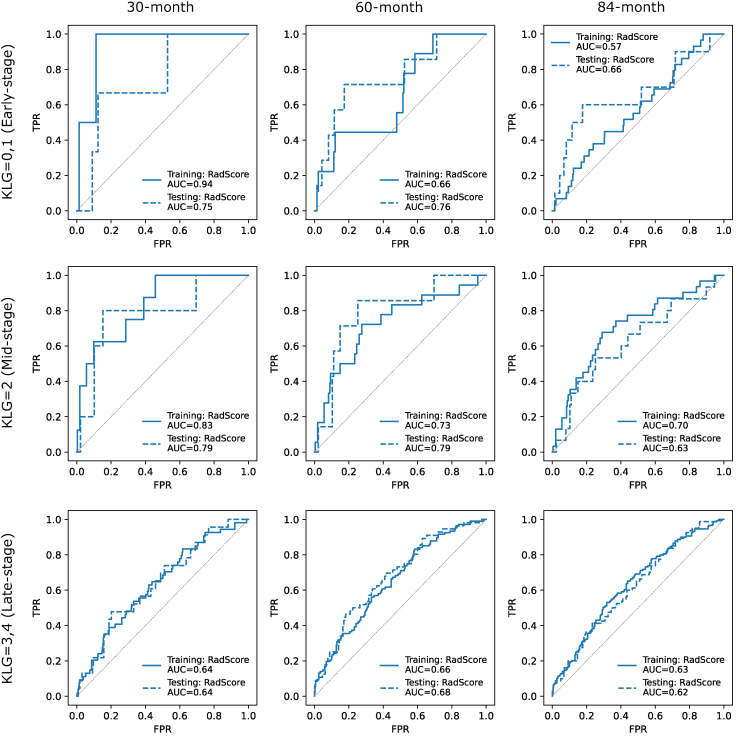
The receiver operating characteristic curves of RadScore in predicting 30-, 60-, and 84-month KR classification in training and testing under different disease stages at baseline visit. High predictive values can be observed within the early-stage (KLG < 2) and mid-stage (KLG ​= ​2) for predicting fast disease progressions (30-/60-month KR).

During subgroup analysis, RadScore demonstrated high predictive values for fast progressors (KR+ (30 ​m)) within early-stage patients (KLG < 2) with AUC ​= ​0.94/0.75 (training/testing) (Fig. 3). It also had high discriminative power for KR at all time points within mid-stage patients (KLG ​= ​2), and the highest AUCs (0.83/0.79) were also achieved in predicting 30-month KR occurrence.

### 3.4 Optimal prognostic performance by KR risk score

The KR risk score achieved the best KR prognosis performance by combining RadScore and KLG. Table 3 reports the performance comparison by C-index and time-dependent AUCs among KLG, RadScore, PFOA ​+ ​KLG, and KR risk score (RadScore ​+ ​KLG). KLG itself had a C-index of 0.83 in training and 0.82 in testing, which were significantly improved by the addition of RadScore to 0.85/0.84 (*p*-value ​= ​0.003/0.002). Similar trends were observed in time-dependent AUCs where the highest was achieved by the KR risk score (30-month: 0.91/0.83, 60-month: 0.89/0.87, 84-month: 0.86/0.86). The KR risk score also achieved significantly higher C-index values than the PFOA ​+ ​KLG model in both the training (*p*-value ​= ​0.035) and testing set (*p*-value ​= ​0.011). Hazard ratios and *p*-values of the covariates of the KR risk score are presented in Table S3.

**Table 3:** Training and testing performance of three knee replacement risk prediction models.

Model	Training		Testing	
	C-index	*p*-value	C-index	*p*-value

KLG	0.83 (0.81–0.86)	**0.003**	0.82 (0.78–0.86)	**0.002**
RadScore	0.78 (0.75–0.81)	**<0.001**	0.78 (0.74–0.82)	**0.018**
PFOA ​+ ​KLG	0.84 (0.81–0.86)	**0.035**	0.82 (0.78–0.86)	**0.011**
KR risk score (RadScore ​+ ​KLG)	0.85 (0.82–0.87)	-	0.84 (0.80–0.87)	-
	30 ​m AUC	*p*-value	30 ​m AUC	*p*-value
KLG	0.90 (0.86–0.92)	**0.007**	0.80 (0.73–0.87)	**0.020**
RadScore	0.83 (0.79–0.88)	**0.015**	0.81 (0.74–0.87)	0.290
PFOA ​+ ​KLG	0.90 (0.87–0.92)	**0.023**	0.81 (0.74–0.88)	0.191
KR risk score (RadScore ​+ ​KLG)	0.91 (0.89–0.94)	-	0.83 (0.77–0.89)	-
	60 ​m AUC	*p*-value	60 ​m AUC	*p*-value
KLG	0.87 (0.85–0.90)	**<0.001**	0.84 (0.79–0.89)	**<0.001**
RadScore	0.82 (0.78–0.85)	**<0.001**	0.83 (0.78–0.87)	0.123
PFOA ​+ ​KLG	0.88 (0.85–0.91)	**0.045**	0.85 (0.79–0.90)	**0.002**
KR risk score (RadScore ​+ ​KLG)	0.89 (0.87–0.92)	-	0.87 (0.83–0.91)	-
	84 ​m AUC	*p*-value	84 ​m AUC	*p*-value
KLG	0.84 (0.81–0.87)	**0.008**	0.84 (0.80–0.87)	**0.003**
RadScore	0.78 (0.74–0.81)	**<0.001**	0.79 (0.74–0.83)	**0.009**
PFOA ​+ ​KLG	0.85 (0.82–0.87)	0.093	0.84 (0.80–0.88)	**0.007**
KR risk score (RadScore ​+ ​KLG)	0.86 (0.83–0.88)	-	0.86 (0.82–0.89)	-

Note: One-sided *p*-values were calculated by permutation test with 1000 iterations. *P*-value less than 0.05 (bolded) was considered significant. Significant performance improvements can be observed when combining RadScore with KLG, compared to using KLG alone.

Abbreviations: C-index, concordance index; RadScore, radiomics score; KLG, Kellgren and Lawrence grade; 30 ​m, 30-month; 60 ​m, 60-month; 84 ​m, 84-month.

### 3.5 Risk stratification and survival analysis

**Fig. 4:**
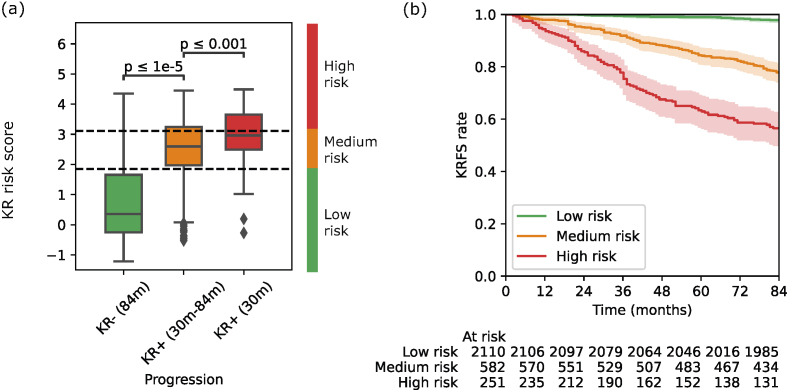
(a) KR risk score (RadScore ​+ ​KLG) distribution comparisons among non-progressors within 84 months (KR- (84 ​m)), slow progressors later than 30 months (KR+ (30 m – 84 ​m)), and fast progressors earlier than 30 months (KR+ (30 ​m)). (b) Knee replacement-free survival (KRFS) curves of the low risk (green), medium risk (orange), and high risk (red) patients from Kaplan-Meier analysis on the entire patient cohort.

Significant KR risk score differences were detected among the three follow-up time points, as shown in Fig. 4(a). The KR risk scores of non-progressive patients at 84 months were the lowest, with an average value of 0.74. They were significantly higher (*p*-value < 0.001) for slow progressors (KR+ (30m–84 ​m)) with an average value of 2.42. Fast progressors (KR+ (30 ​m)) achieved the highest average KR risk score (2.96), which is significantly higher than slow progressors (*p*-value < 0.001).

**Fig. 5:**
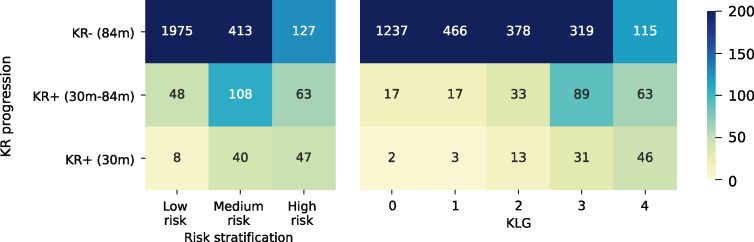
Confusion matrix of the proposed stratification system and KLG in predicting the three KR progression speeds.

Three risk groups were stratified based on the optimised KR risk score thresholds of 1.86 and 3.11 (Fig. 4(a), dashed lines), and distinct survival patterns were observed among the three risk groups Fig. 4(b). Patients with KR risk score less than 1.86 were classified as low risk (n ​= ​2110) with minimum risk of KR progression within 84 months (6%), as drawn by the survival curves in Fig. 4(b) and confusion matrix in Fig. 5. Meanwhile, patients with the score of more than 1.86 but less than 3.11 were classified as medium risk (n ​= ​582), showing a relatively higher risk of KR within 84 months (25%), but the fast progression (KR+ (30 ​m)) rate remained as low as 7%. The high-risk group of patients (n ​= ​251) who had the score greater than 3.11 demonstrated the highest risk of receiving KR within 84 months (48%) and 30 months (19%). In contrast, only 11% and 3% of KLG ​= ​2 patients were slow and fast progressors, respectively. Although similar rates of slow (49%) and fast (20%) progressors were achieved by the KLG of 4, more progressive patients were identified by the proposed high-risk criteria. Specifically, the positive predictive value (PPV) of our RadScore for predicting knee replacement (KR) was 46.41%, significantly outperforming the KLG's PPV of 34.54%. Furthermore, in predicting KR within 30 months, the RadScore's PPV was 27.01%, compared to only 11.61% for the KLG. These findings underscore the enhanced precision of RadScore over KLG in identifying patients at high risk of both overall KR and rapid progression to KR within a shorter timeframe.

## 4 Discussion

This study, for the first time, highlights the importance of quantitative analysis of PF joint from lateral knee radiographs in KR prediction. It also provides a comprehensive tool incorporating TF and PF joints radiographic information for assisting clinicians in stratifying patients based on disease progression speed. The developed PF joint RadScore was validated as an independent prognostic factor for KR and achieved better KR prognostic performance in early- and mid-stage. The comprehensive KR risk score achieved the highest performance based on the combination of two joints’ radiographic information. Distinct KR-free survival patterns were delineated for the three stratified risk groups, which could benefit precise rehabilitation therapy by prioritising higher risk patients with faster disease progressions.

### 4.1 Clinical implications

Despite the heterogeneous KR prediction performance, all three regional ROIs of PF joint demonstrated certain prognostic values. Those ROIs are located at the surface between the patella and the femoral notch, known as the trochlea, which is a key area of contact between these bones. According to Wolff's Law, bones adapt to the loads under which they are placed. Therefore, changes in this area can reflect the abnormal stresses on the knee, indicating early signs of OA. Previous research by Bayramoglu et al. once emphasised the importance of ROI location [35]. It confirmed the PFOA diagnostic ability of two lateral patella ROIs at the PF joint margin [36], which was consistent with the ROI definitions in our study. The best-performing ROI was located on the inferior region of the PF joint, with a significant area outside the patella bone. Based on the distinctive patella shape differences observed from the three groups of patients, the final RadScore, built mainly from first-order radiomic features, may capture the patella morphological change due to the altered mechanical loading with knee joint deterioration. Similarly, previous studies have suggested that patella shape and alignment strongly correlate with PFOA, PF joint cartilage defect, and physical activity reduction [[37], [38], [39], [40], [41]]. Such visually appreciable changes were effectively captured and quantified by radiomics, which might reduce the inter-observer variability and improve diagnostic consistency.

### 4.2 Independent predictors of KR

Results from multivariate analysis suggest that our radiomic characterisation of PF joint on lateral radiographs (RadScore) was independently prognostic to the TF joint KLG, and the integration of RadScore to KLG could significantly boost the performance of KR prediction in the MOST dataset. Despite the limited increments in C-index and AUC values in the entire MOST cohort, our model revealed its unique advantages in predicting fast progressors among early- and mid-stage patients in the subgroup analysis. In contrast, the study that primarily focused on the TF joint demonstrated the highest performance for late-stage patients [7]. This is consistent with previous research conclusions indicating that the PFOA manifests before the TFOA [15]. Predicting fast progressors in the early and mid stages is crucial, as early intervention may alter the disease trajectory and lead to improved outcomes.

On the other hand, demographic information had limited independent prognostic values for KR prediction, and the current clinical diagnostic criteria for the PF joint (PFOA) did not achieve an independent prognostic value. This finding further underlies the importance of PF joint as well as its quantitative characterisation compared to the other risk factors. It may also suggest stronger correlations of PF joint with symptomatic presentations, which is consistent with previous clinical observations [17].

### 4.3 Limitations of this study

Several limitations in this study in data interpretation shall be fully aware, which warrant further improvements in future investigations. First, only the initial visit radiographs were analysed for KR prediction. A dynamic risk assessment method using image sets from a time series may further improve prediction accuracy. Second, although the MOST dataset is combined by several cohorts, a comprehensive assessment of the proposed patella RadScore and KR risk score on various external datasets with different patient distributions is necessary to further demonstrate the model's generalizability. We have investigated the OAI dataset, but it cannot fulfil our purposes. The dataset lacks sufficient subjects with lateral view X-rays; none of these cases had KR surgery records. Future research could explore alternative datasets or await updated data releases. Third, our machine learning analysis of lateral view radiographs requires patella segmentation, which was achieved by manually contouring. In addition, the KLG of the PA view was acquired by manual reading. A fully automated risk assessment pipeline requires automatic lateral view patella segmentation and quantitative TF and PF joint assessments from the PA view radiographs, which will be conducted in the next stage of our research.

## 5 Conclusion

In summary, we developed a PF joint RadScore on lateral knee radiographs, which was validated as an independent prognostic factor to predict KR risk among KOA patients. The KR risk score that incorporates TF and PF joints radiographic information achieved the best KR prognostic performance. Based on this score, the stratification system could triage KOA patients into three distinct KR-free survival groups to reflect the progress speed. It would serve as a clinical reference to guide exercise or other physical therapy for secondary prevention of KOA deterioration.

## Author contributions

Jiang Zhang contributed to the conception and design of the study, analysis and interpretation of the data, and drafting of the article. Tianshu Jiang, Lok-Chun Chan, Sing-Hin Lau, and Wei Wang contributed to the acquisition and preprocessing of the data, logistic support, and critical revision of the article for important intellectual content. Xinzhi Teng provided statistical expertise and critical revision of the article. Ping-Keung Chan, Jing Cai, and Chunyi Wen contributed to the conception and design of the study and critical revision of the article. Chunyi Wen also provided administrative and logistic support and funding for this study. All authors gave final approval of the version to be submitted.

## Funding

This study is supported by SZRI start-up (“百城百園”專項啟動基金", I2021A013) and RISports Seed Fund (P0043526), Projects of RISA (P0043001, P0043002), Project of RIAM (P0041372), and Project of Strategic Importance Fund (P0035421) of the 10.13039/501100004377Hong Kong Polytechnic University.

## Code availability

The code can be downloaded publicly at https://github.com/John136219655/PatellaRadiomics. Other information is available upon request to the corresponding author.

## Conflict of interest

The authors have no conflicts of interest relevant to this article.
