# Supplementary material for: Radiomics analysis of patellofemoral joint improves knee replacement risk prediction: Data from the Multicenter Osteoarthritis Study (MOST)
Source: Osteoarthr Cartil Open. 2024 Feb 24;6(2):100448. doi: 10.1016/j.ocarto.2024.100448 (PMC10910336; doi:10.1016/j.ocarto.2024.100448)
Supplement: Multimedia component 1 [file mmc1.docx]

**Supplementary Material**

**Participants**

The Multicenter Osteoarthritis Study (MOST) recruited senior subjects aged from 50 to 79 who had pre-existing or a higher risk of in the community. Subjects from the MOST received initial diagnosis in clinic between April, 2003 and April, 2005 and were followed up at 15-, 30-, 60-, 72-, and 84-month by telephone interview or clinical visit, depending on the reported knee symptoms and random selection.

**Variable Acquisition**

The Kellgren-Lawrence grade (KLG) and radiographic patella-femoral osteoarthritis (PFOA) status were independently given by two readers from fixed-flexion posteroanterior and weight-bearing lateral radiographs. Score values were adjusted upon the occurrence of meaningful discrepancies until a consensus was reached.

The time and event of the total knee replacement from the initial visit was determined by the medical records, knee x-rays, or self-report. Medical records were reviewed by a physician adjudicator.

Detailed definitions of the variables used in this study can be found in the MOST dataset descriptions (<https://agingresearchbiobank.nia.nih.gov/studies/most/>).

Table S1. Image preprocessing and feature extraction parameters.

| Parameter | Value |
| --- | --- |
| Normalization scale | 100 |
| Pixel value offset | 600 |
| Pixel value thresholding | 0-1200 |
| Resample pixel size (mm) | [0.5,0.5] |
| Image/mask interpolation algorithm | Nearest neighbor |
| Mask partial volume threshold | 0.5 |
| Interpolation grid alignment | Align grid origins |
| Gray-level discretization bin number | 32 |
| Image filters | Unfiltered, Laplacian-of-Gaussian, Wavelet |
| Kernel size of Laplacian-of-Gaussian filter (mm) | [1,2,3] |
| Wavelet filter type | Coilf1 |
| Wavelet filter decompositions | [LL,HL,LH,HH] |
| Feature class | First-order, GLCM, GLRLM, GLSZM, GLDM, NGTDM |

Note: For a detailed description and comprehensive list of all radiomic features analyzed in this study, readers are directed to the PyRadiomics documentation available at https://pyradiomics.readthedocs.io/en/latest/features.html. This documentation provides extensive information on each feature's calculation and theoretical background, ensuring thorough understanding and facilitating the reproducibility of our analyses.

Table S2. Details of the selected radiomic features and model coefficients of the final patella RadScore.

| Alias | Image | Class | Name | Mean coefficient | Normalization mean | Normalization scale |
| --- | --- | --- | --- | --- | --- | --- |
| F1 | Original | First Order | Mean | 0.199 | 555.43 | 65.32 |
| F2 | Original | NGTDM | Strength | -0.160 | 16.34 | 7.67 |
| F3 | LoG (sigma=2mm) | GLRLM | ShortRunHighGrayLevelEmphasis | -0.090 | 292.31 | 46.85 |
| F4 | Wavelet (LH) | GLCM | ClusterShade | -0.097 | -35.24 | 33.18 |
| F5 | Wavelet (LL) | First Order | Mean | 0.198 | 1110.87 | 130.63 |
| Intercept |  |  |  | --0.24 |  |  |
| Note: RadScore can be calculated by the linear combination of the 10 radiomic features plus the intercept: $RadScore=\sum_{i} {(f}_{i}-m_{i})/s_{i}\cdot c_{i}+a$, where $f_{i}$ is value of the ith feature (Fi), $m_{i}$ is the normalization mean, $c_{i}$ is the normalization scale, $c_{i}$ is the mean coefficient, and $a$ is the intercept. | | | | | | |

Table S3. Coefficients of the KR risk score built by multivariate Cox regression.

| Covariate | Hazard ratio (95% confidence interval) | *p*-value |
| --- | --- | --- |
| RadScore | 1.49 (1.27-1.74) | < 0.001 |
| KLG | 1.94 (1.70-1.23) | < 0.001 |
